# Research for association and correlation between stress at workplace and individual mental health

**DOI:** 10.3389/fpubh.2024.1439542

**Published:** 2024-11-22

**Authors:** Mi-Jeong Lee, Wanhyung Lee

**Affiliations:** ^1^Graduate School of Gachon University in Public Health, Incheon, Republic of Korea; ^2^Department of Nursing, Andong Science College, Andong, Republic of Korea; ^3^Department of Preventive Medicine, College of Medicine Chung-Ang University, Seoul, Republic of Korea

**Keywords:** job stress, depression, KOSS-SF, CES-D, PHQ-9, manufacturing sector

## Abstract

**Background:**

Occupational stress negatively affects mental health and productivity. Managing worker mental health has been equated to assessing workplace stressors, although there are challenges in screening and managing the mental health of vulnerable workers. This study aimed to determine the correlation between workplace stress characteristics and two depression assessment questionnaires to guide workplaces on assessing job stress more effectively.

**Methods:**

A cross-sectional survey study was conducted with 812 workers from manufacturing sector in Korea. Depression was measured using the CES-D and PHQ-9 questionnaires, and the KOSS-SF was used to assess job stress.

**Results:**

The results showed that 26.2% of participants had high job stress levels, with job control and demands being the highest subfactors. The CES-D and PHQ-9 were positively correlated with job stress factors, with the PHQ-9 showing stronger correlations than the CES-D.

**Conclusion:**

The CES-D and PHQ-9 were associated with workplace stress; however, the CES-D was not associated with job control, whereas the PHQ-9 was not associated with job demands or relationship conflicts. Additionally, the PHQ-9 showed a higher correlation with workplace stress than the CES-D. The correlation between depression screening tests differed according to workplace stress characteristics, but the PHQ-9 was helpful in screening workers for depression.

## Background

Occupational stress is stress from the workplace or organization, defined by the National Institute for Occupational Safety and Health as “harmful physical and emotional reactions that occur when job demands are inconsistent with a worker’s abilities, resources, and desires” (1). In 2009, the International Social Survey Program released data on the rate of job stress, which means “feeling stressed at work,” by country; the rate of job stress was 87% in Korea, higher than the Organization for Economic Cooperation and Development average of 78%. Compared to 78% in the U.S. and 72% in Japan, job stress in Korea is exceptionally high. A study by Chang et al. (2) surveyed 6,977 workers in 254 businesses and found that 5% were in the healthy group, 73% were in the potentially stressed group, and 22% were in the high-risk stressed group (2).

Job stress leads to job dissatisfaction, turnover, absenteeism, and decreased productivity (3, 4), which not only causes economic losses through accidents, deaths, and lost wages but also physically increases the prevalence of cardiovascular diseases and diabetes and affects mental health by causing conditions such as depression and anxiety (5, 6). In particular, depression has been shown to increase the frequency of depressive symptoms by approximately four times compared with job stress (7). Depression in workers not only increases the risk of cardiovascular diseases but also leads to psychiatric side effects such as drug abuse, alcoholism, and suicide (8). Therefore, it is very important to prevent and manage depression in advance. According to statistics from the World Health Organization, approximately 3.8% of the population in worldwide experiences depression, and approximately 280 million people suffer from depression worldwide. In Korea, depression screening was introduced in 2018 as part of a national health checkup for employed members of the National Health Insurance to reduce suicide risk. There are various strategies for preventing severe mental health problems, including suicide, in Korean workplaces (9). According to Article 5 (2) of the Occupational Safety and Health Act, the current system for managing workers’ occupational stress in Korea requires employers to “create a pleasant working environment and improve working conditions to reduce physical fatigue and mental stress.”

According to a previous report, structural interventions are based on the principle of prevention, introducing methods to alleviate stress factors through education on basic mental health management as the primary prevention technique and employing screening using mental health examinations for workers as the secondary prevention method (10). Most large-sized enterprises establish mental health programs to manage psychological wellness among their employees, but it is difficult for small-and medium-sized enterprises to achieve this due to a lack of cost and interest. Therefore, for job stress management, health managers or health management-entrusted organizations at workplaces use the Korean Job Stress Measurement Tool to evaluate job stress and manage it according to the results of the Guide (H-67-2022), a guideline for measuring job stress factors created and distributed by the Korea Occupational Safety and Health Administration.

The Korean version of the tool does not include items on personal characteristics or stressors other than work and has limitations in that it does not measure symptom levels as a result of stress, but rather assesses job stress factors and interprets the results by the workplace unit rather than by the individual (2).

To date, there has been few studies on the relationship of stress characteristics at the workplace and which questionnaires are suitable for measuring individual mental health among workers. Therefore, this study was conducted to determine the extent to which the Center for Epidemiological Studies-Depression Scale (CES-D) and the Patient Health Questionnaire-9 (PHQ-9) questionnaires, which are relatively popular depression assessment tools used in primary health care, correlate better with workplace stress according to domains of the Korean Occupational Stress Scale-Short Form (KOSS-SF).

## Methods

### Study participants and data collection

This study was conducted from February to August 2022. After obtaining consent from workers of small-and medium-sized manufacturing businesses that receive healthcare entrusted to them by the health management specialist institution of a hospital in Incheon, the purpose of the study and how to complete the questionnaire were explained. Before the self-completion questionnaires were received, assurance was provided that the contents of the questionnaires will be used only for the study. Data were collected by asking workers at high-risk workplaces in healthcare consignment sites to complete the KOSS-SF and CES-D questionnaires. The KOSS-SF and PHQ-9 questionnaires were distributed to the health workers in charge of the workplace and submitted on the day of the medical examination. A total of 647 workers (565 men and 82 women) responded to the KOSS-SF and CES-D, while 165 workers (125 men and 40 women) responded to the KOSS-SF and the PHQ-9 depression questionnaires.

After excluding 20 individuals who did not consent to the use of their results at the time of screening or who responded insufficiently to the survey questions, data from 812 workers were included in the final analysis.

### Main variables

#### Occupational stress assessment

Occupational stress was assessed using a shortened version of the KOSS-SF (2). The KOSS-SF has a basic form consisting of a questionnaire with 43 items in eight areas and a short form consisting of 24 items in seven areas after factor analysis and validity testing. This study used a short form that can identify important sources of stress among Korean workers that can be easily applied in the field. For each question, the respondents were asked to answer on a 4-point scale of “not at all,” “not true,” “true,” “yes,” and “very true.” Items with higher scores were given 1–4 points, and items with lower scores were reverse-coded from 4 to 1 points. We obtained a scaled score for each area by using the scoring method proposed by the developers, with higher scores indicating higher job stress.

The scores were calculated as follows;


$$\begin{array}{l} \mathrm{Scaled score for each area = (actual score - number of}\;\\ \mathrm{questions)}\times \;\mathrm{100/(highest possible score - number of}\;\\ \mathrm{questions) = Job Stress Total Score = (sum of scaled}\;\\ \mathrm{scores in each of the seven domains)}/7 \end{array}$$

The gender reference value indicates the actual score of the target employees and the quartile of national employees, with the top 50% as high and the bottom 50% as low.

### Depression

#### CES-D

The current study used a Korean version of the CES-D developed by Radloff (11) and translated by Cho and Kim (12). The CES-D is a self-reported depression scale consisting of 20 items including depressed mood, positive emotions, physical symptoms, sluggish behavior, and interpersonal factors. Each item measures the frequency of experience in the past week as “extremely rarely,” “sometimes,” “often,” and “most of the time” on a 4-point scale, and the positive items are reverse-scaled so that a higher mean score indicates higher depression. In this study, the 21 points suggested by a previous study (12) were used as cutoff points to determine depression, with 0–20 points indicating normal and 21 points or more indicating depressed.

#### PHQ-9

The Korean version (13) of the PHQ-9 developed by Spitzer et al. (14) was used for measurement. The PHQ is a self-administered questionnaire developed to promote the recognition and diagnosis of common mental disorders in primary care settings. Among them, the PHQ-9, which consists of nine questions for the diagnosis of major depressive disorder, measured the frequency of symptom occurrence in the past 2 weeks on a 4-point scale, with 0 for “never,” 1 for “several days,” 2 for “more than a week,” and 3 for “almost every day.” The sum was calculated to determine the depressive state if the total score was 10 or more out of 27 points or if the score of item 9 was 1 or more (13).

### Statistical analysis

We conducted the following analyses. Owing to the differing KOSS criteria for men and women, all results were analyzed by considering this difference between the two sexes.

1. The general characteristics and job stress levels of the participants were statistically processed as means and percentages.
2. The CES-D and PHQ-9 levels according to general characteristics and job stress were analyzed using the Chi-squared test.
3. The relationships between job stress and CES-D and PHQ were analyzed using multiple logistic regression.
4. The association between job stress and CES-D and PHQ was analyzed using the Pearson correlation coefficient.
5. Statistical significance was set at *p* < 0.05. All statistical analyses were conducted using SAS version 9.4 (SAS Institute, Cary, NC, USA).

## Results

The demographic information and job stress levels are shown in Table 1. In total, 812 respondents participated in the survey. A total of 418 (51.5%) were aged 20 to 39 years, 380 (56.8%) were aged 40 to 59 years, and 14 (1.7%) were aged 60 years or older. There were 690 (85.0%) males and 122 (15.0%) females, with 518 (63.8%) working production jobs and 294 (36.2%) working office jobs. In terms of job stress factors, 213 (26.2%) of the participants had a total score of 213, 247 (30.4%) had job demands, 353 (43.5%) had job autonomy, 242 (29.8%) had relationship conflicts, 137 (16.9%) had job insecurity, 230 (28.3%) had organizational systems, 182 (22.4%) had inadequate compensation, and 138 (17.0%) had workplace culture, with the highest number being job autonomy factors and the lowest number being job insecurity factors.

**Table 1:** General characteristics and occupational stress according to the KOSS-SF.

Variable	Categories	*N* (%)
Characteristics		
Age	20 ~ 39	418 (51.5%)
	40 ~ 59	380 (56.8%)
	>60	14 (1.7%)
Sex	Male	690 (85.0%)
	Female	122 (15.0%)
Occupational classification	White-collar	518 (63.8%)
	Blue-collar	294 (36.2%)
KOSS-SF domains		
Total score	Low	599 (73.8%)
	High	213 (26.2%)
Job demand	Low	565 (69.6%)
	High	247 (30.4%)
Job control	Low	459 (56.5%)
	High	353 (43.5%)
Interpersonal conflict	Low	570 (70.2%)
	High	242 (29.8%)
Job insecurity	Low	675 (83.1%)
	High	137 (16.9%)
Organization system	Low	582 (71.7%)
	High	230 (28.3%)
Lack of reward	Low	630 (77.6%)
	High	182 (22.4%)
Occupational climate	Low	674 (83.0%)
	High	138 (17.0%)

The CES-D scores according to the participants’ general characteristics and job stress factors are shown in Table 2. A cross-tabulation analysis was conducted to examine the CES-D level according to general characteristics and job stress factors. A total of 647 participants responded to the KOSS-SF and CES-D questionnaires, of whom 602 (93.0%) scored as CES-D-normal and 45 (7.0%) were high-risk. In terms of age, out of those aged 20–39 years, 335 (92.3%) were normal and 28 (7.7%) were at risk; out of those aged 40–59 years, 255 (94.1%) were normal and 16 (5.9%) were at risk; and out of those aged 60 years and older, 12 (92.3%) were normal and 1 (7.7%) was at risk, with the highest proportion of those aged 20–39 years considered at risk of depression. In terms of sex, 523 males (92.6 per cent) were normal and 42 (7.4 per cent) were high-risk for depression, while 79 females (96.3 per cent) were normal and 3 (3.7 per cent) were high-risk for depression, with males having a higher proportion of high-risk depression, though this was not statistically significant (*p* = 0.20). In terms of occupation, 252 (91.3%) office workers were normal and 24 (8.7%) were high-risk; 350 (94.3%) were normal and 21 (5.7%) were high-risk, with a higher proportion of high-risk depression among office workers; however, this was not statistically significant (*p* = 0.13).

**Table 2:** General characteristics and domains of KOSS-SF according to depression by the CES-D.

		Depression by CES-D, N(%)		*p*-value
		No	Yes	
No. of participants		602 (93.0)	45 (7.0)	
Age (years)				0.4450
20 ~ 39		335 (92.3)	28 (7.7)	
40 ~ 59		255 (94.1)	16 (5.9)	
>60		12 (92.3)	1 (7.7)	
Sex				0.2096
Male		523 (92.6)	42 (7.4)	
Female		79 (96.3)	3 (3.7)	
Occupational classification				0.1337
White-collar		252 (91.3)	24 (8.7)	
Blue-collar		350 (94.3)	21 (5.7)	
KOSS-SF domains				
Total score	Low	477 (95.8)	21 (4.2)	<0.0001
	High	125 (83.9)	24 (16.1)	
Job demand	Low	419 (95.7)	19 (4.3)	0.0002
	High	183 (87.6)	26 (12.4)	
Job control	Low	379 (94.5)	22 (5.5)	0.0610
	High	223 (90.66)	23 (9.4)	
Interpersonal conflict	Low	440 (94.2)	27 (5.78)	0.0589
	High	162 (90.0)	18 (10.0)	
Job insecurity	Low	522 (94.7)	29 (5.26)	<0.0001
	High	80 (83.3)	16 (16.7)	
Organization system	Low	456 (95.2)	23 (4.8)	0.0003
	High	146 (86.9)	22 (13.1)	
Lack of reward	Low	496 (95.0)	26 (5.0)	<0.0001
	High	106 (84.8)	19 (15.2)	
Occupational climate	Low	513 (94.5)	30 (5.5)	0.0011
	High	89 (85.6)	15 (14.4)	

The statistically significant factors were the total score, job demands, job autonomy, organizational system, inadequate compensation, and workplace culture. The total score was 447 (95.8%) normal and 21 (4.2%) high-risk in the low-risk group, and 125 (83.9%) normal and 24 (16.1%) high-risk in the high-risk group, with a higher proportion of high-risk in the high-risk group, which was statistically significant (*p* < 0.0001). The job demands score was 419 (95.7%) normal and 219 (4.3%) high-risk in the low-risk group and 182 (87.6%) normal and 26 (12.41%) high-risk in the high-risk group, with a higher proportion of high-risk in the high-risk group, which was statistically significant (*p* = 0.0002). The job insecurity score was 522 (94.2%) normal and 29 (5.26) high-risk in the low-risk group and 80 (83.3%) normal and 16 (16.7%) high-risk in the high-risk group; the proportion of high-risk was significantly higher in the high-risk group (*p* = 0.001). The organizational system score was 456 (95.2%) normal and 23 (4.8) high-risk in the low-risk group and 146 (86.9%) normal and 22 (13.1%) high-risk in the high-risk group; the proportion of high-risk in the high-risk group was higher and statistically significant (*p* = 0.003). The compensation inadequacy score was 496 (95.20%) normal and 26 (5.0%) high-risk in the low-risk group and 106 (84.8%) normal and 19 (15.2%) high-risk in the high-risk group; the proportion of high-risk in the high-risk group was higher and statistically significant (*p* = 0.001). The organizational culture score was 513 (94.5%) normal and 30 (5.5%) high-risk in the low-risk group and 89 (85.6%) normal and 15 (14.4%) high-risk in the high-risk group; the proportion of high-risk in the high-risk group was higher and statistically significant (*p* = 0.001).

The PHQ levels of the participants according to their general characteristics and job stress factors are shown in Table 3. A cross-tabulation analysis was conducted to examine the PHQ-9 level according to general characteristics and job stress factors. A total of 165 participants responded to the KOSS-SF and PHQ questionnaires, of whom 140 (84.4%) were PHQ-normal and 25 (15.1%) were high-risk. Regarding age, 41 (74.5%) were normal and 14 (25.5%) were at risk in the 20–39 years age group, 98 (89.9%) were normal and 11 (10.1%) were at risk in the 40–59 years age group, and 1 (100%) was normal and 0 (o%) was at risk in the 60 years and above age group, with a higher proportion of high-risk in the 20–39 years age group, which was not statistically significant (*p* = 0.09). In terms of sex, 101 (80.8%) were normal and 24 (19.2%) were high-risk, while 39 (97.5%) were normal and 1 (2.5%) was high-risk, with males having a higher proportion of high-risk, which was statistically significant (*p* = 0.01). Regarding occupation, 13 office workers were (72.2%) normal and 5 (27.8.7%) were high-risk, and 118 (80.3%) non-office workers were normal and 29 (19.7%) were high-risk, with a significantly higher proportion of high-risk office workers (*p* = 0.03).

**Table 3:** General characteristics and domains of KOSS-SF according to depression by the PHQ-9.

		Depression by PHQ-9, N(%)		*p*-value
		No	Yes	
No. of participants		140 (84.4)	25 (15.1)	
Age (years)				0.009
20 ~ 39		41 (74.5)	14 (25.5)	
40 ~ 59		98 (89.9)	11 (10.1)	
>60		1 (100)	0 (0.0)	
Sex				0.0101
Male		101 (80.8)	24 (19.2)	
Female		39 (97.5)	1 (2.5)	
Occupational classification				<0.0001
White-collar		13 (72.2)	5 (27.8)	
Blue-collar		118 (80.3)	29 (19.7)	
KOSS-SF domains				
Total score	Low	95 (94.0)	6 (29.7)	<0.0001
	High	45 (70.3)	19 (29.7)	
Job demand	Low	113 (88.9)	14 (11.0)	0.0002
	High	27 (71.0)	11 (28.9)	
Job control	Low	52 (89.7)	6 (10.3)	0.0610
	High	88 (82.2)	19 (17.8)	
Interpersonal conflict	Low	90 (80.7)	13 (12.6)	0.0589
	High	50 (80.7)	12 (19.3)	
Job insecurity	Low	112 (90.3)	12 (9.7)	<0.0001
	High	28 (68.3)	13 (31.7)	
Organization system	Low	94 (91.3)	9 (8.7)	0.0003
	High	46 (74.2)	16 (25.8)	
Lack of reward	Low	99 (91.7)	9 (8.3)	<0.0001
	High	41 (71.9)	16 (28.0)	
Occupational climate	Low	118 (90.1)	13 (9.9)	0.0011
	High	22 (64.7)	12 (35.3)	

The statistically significant factors were the total score, job demands, job autonomy, organizational system, compensation inadequacy, and workplace culture. The total score was 95 (94.0%) normal and 6 (29.7%) high-risk in the low-risk group and 45 (70.3%) normal and 19 (29.7%) high-risk in the high-risk group; the proportion of high-risk in the high-risk group was significantly higher (*p* = 0.001). Job demand scores were 113 (70.3%) normal and 14 (11.0%) high-risk in the low-risk group, and 27 (71.0%) normal and 11 (28.91%) high-risk in the high-risk group, with a higher proportion of high-risk in the high-risk group that was statistically significant (*p* = 0.007). The job insecurity score was 112 (90.3%) normal and 12 (9.7%) high-risk in the low-risk group, and 28 (68.3%) normal and 13 (31.7%) high-risk in the high-risk group; the proportion of high-risk in the high-risk group was higher and statistically significant (*p* = 0.0007). The organizational system score was 94 (91.3%) normal and 9 (8.7%) high-risk in the low-risk group, and 46 (74.2%) normal and 16 (25.8%) high-risk in the high-risk group, with a higher proportion of high-risk in the high-risk group, which was statistically significant (*p* = 0.003). The compensation inadequacy score was 99 (91.7%) normal and 9 (8.3%) high-risk in the low-risk group and 41 (71.9%) normal and 16 (28.0%) high-risk in the high-risk group, with a higher proportion of high-risk in the high-risk group, which was statistically significant (*p* = 0.0008). The occupational climate score was 118 (90.1%) normal and 14 (9.9%) high-risk in the low-risk group and 22 (64.7%) normal and 12 (35.3%) high-risk in the high-risk group; the proportion of high-risk in the high-risk group was higher and statistically significant (*p* = 0.0002).

Logistic regression analyses were performed to adjust for confounding variables such as age, sex, and occupational classification. The odds ratio (OR, 95% confidence interval [CI]) for depression according to the CES-D scores was calculated, and the high total score of the KOSS-SF was 4.83 (2.56–9.10). The high level of job demands was 2.95 (1.57–5.55) and the high level of job control was 2.08 (1.11–3.89) in the depression group. The depression group according to the CES-D showed a higher risk for a high level of relationship conflict (2.06 [1.07–3.95]), job insecurity (4.14 [0.35–16.64]), organizational structure (3.2 [1.7–6.04]), compensation inadequacy (3.89 [2.03–7.41]), and occupational climate (3.02 [1.53–5.04]) than the non-depression group, respectively. The depression group according to the PHQ-9 also showed an increased risk of high levels of each job stress item. Specifically, a higher risk for a high level of job stress was seen with a total score of 7.98 (2.70–23.56), 4.33 (1.65–11.40) for job insecurity, 4.14 (1.59–10.74) for organizational system, 5.09 (1.97–13.18) for compensation inadequacy, and 7.28 (2.65–19.99) for occupational climate, respectively (Table 4).

**Table 4:** The risk of depression according to the level of KOSS-SF by multiple logistic regression.

Odd Ratio (95% confidence intervals)			
KOSS-SF domains		CES-D	PHQ-9
Total score	Low	Reference	Reference
	High	4.83 (2.56–9.10)	7.98 (2.70–23.56)
Job demand	Low	Reference	Reference
	High	2.95 (1.57–5.55)	2.45 (0.98–6.14)
Job control	Low	Reference	Reference
	High	2.08 (1.11–3.89)	2.31 (0.82–6.52)
Interpersonal conflict	Low	Reference	Reference
	High	2.06 (1.07–3.95)	1.66 (0.68–4.05)
Job insecurity	Low	Reference	Reference
	High	4.14 (0.35–16.64)	4.33 (1.65–11.40)
Organization system	Low	Reference	Reference
	High	3.20 (1.71–6.00)	4.14 (1.59–10.74)
Lack of reward	Low	Reference	Reference
	High	3.89 (2.03–7.41)	5.09 (1.97–13.18)
Occupational climate	Low	Reference	Reference
	High	3.02 (1.53–5.94)	7.28 (2.65–19.99)

All results were adjusted by age, sex, and occupational classification.

A correlation analysis was conducted between job stress and depression (Table 5). The CES-D score was positively correlated, with a higher total score for job stress being statistically associated with higher depression (r = 0.41, *p* < 0.0001). When analyzed by the job stress subdomains, there were significant positive correlations with job demands (r = 0.31, *p* < 0.0001), job autonomy (r = 0.15, *p* < 0.0001), relationship conflict (r = 0.19, *p* < 0.0001), job insecurity (r = 0.35, *p* < 0.0001), organizational system (r = 0.33, *p* < 0.0001), inadequate compensation (r = 0.32, *p* < 0.0001), and occupational climate (r = 0.29, *p* < 0.0001). Job stress and the PH-9 were positively correlated, with higher total job stress scores being statistically associated with higher levels of depression (r = 0.47, *p* < 0.0001). When analyzed by the sub-domains of job stress, there were significant positive correlations with job demands (r = 0.4, *p* < 0.0001), relationship conflict (r = 0.19, *p* = 0.01), job insecurity (r = 0.46, *p* < 0.0001), organizational systems (r = 0.3, *p* < 0.0001), compensation inadequacy (r = 0.35, *p* < 0.0001), and occupational climate (r = 0.19, *p* = 0.01).

**Table 5:** Correlations between KOSS-SF domains and depression (CES-D/PHQ-9).

Correlation coefficient (*p*-value)		
KOSS-SF	CES-D	PHQ-9
Total score	0.41 (<0.0001)	0.47 (<0.0001)
Job demand	0.31 (<0.0001)	0.40 (<0.0001)
Job control	0.15 (<0.0001)	0.01 (0.99)
Interpersonal conflict	0.19 (<0.0001)	0.19 (0.01)
Job insecurity	0.35 (<0.0001)	0.46 (<0.0001)
Organization system	0.33 (<0.0001)	0.30 (<0.0001)
Lack of reward	0.32 (<0.0001)	0.35 (<0.0001)
Occupational climate	0.29 (<0.0001)	0.20 (0.01)

## Discussion

This study examined the correlation between tools for assessing job stress in organizations and mental health assessment tools for individuals. The KOSS-SF was compared to the CES-D and PHQ-9, which are screening instruments for major depression.

The results showed that 213 (26.2%) of the 812 participants in the survey had high levels of job stress, and the highest percentage of sub-factors were job autonomy (247 [43.5%]) and job demands (30.4%). This was higher than the 22% reported in the high-risk stress group (2). Job stress not only physically increases the prevalence of cardiovascular disease and diabetes but also affects mental health, such as causing depression and anxiety, with the frequency of depressive symptoms increasing fourfold as job stress increases (7). The risk of depression among all workers who responded to the KOSS-SF, CES-D, and PHQ-9 questionnaires was 7.0 and 15.1%, respectively. This was significantly higher than the 3.3% of the population classified as being at risk of depression (15).

Screening for depression based on the CES-D showed that higher KOSS-SF total scores and most of the seven items, except for job insecurity, were associated with a significantly higher risk of depression. Additionally, the correlation analysis showed that the KOSS-SF and CES-D scores were significantly positively correlated in all domains, with the highest correlation being the total score.

This is consistent with previous studies indicating that job stress contributes to an increased risk of depression (16–18). In addition, higher job stress was significantly associated with higher depression, and job demands, relationship conflicts, job insecurity, organizational systems, and compensation inadequacy were significantly and positively related to depression according to the total score and subdomains of job stress (19).

The results of the depression screening based on the PHQ-9 showed that the higher the total score of the KOSS-SF and the seven items, job insecurity, organizational system, lack of reward, and occupational climate, the higher the risk of depression. Correlation analysis showed that the KOSS-SF and PHQ-9 were significantly positively correlated in all areas except for the total score and job control among the seven items, with the highest correlation being the total score. This is similar to the results of the previous study, which showed a significant correlation between the KOSS-SF and PHQ-9 (20).

In summary, the CES-D was associated with all factors of job stress, except job autonomy, and was correlated with all factors. In addition, the PHQ-9 was associated with all job stress factors except job demands and relationship conflicts, correlated with all factors except job autonomy, and had higher correlations than the CES-D.

A previous study found that repeatedly experiencing high job stress was associated with a risk of high levels of depressive symptoms in both men and women (18). Likewise, a study on job stress and depression in female workers also found work-related stress associated with depression and anxiety (21).

According to the World Health Organization statistics, approximately 3.8% of the Korean population experiences depression, and approximately 280 million people suffer from depression worldwide. In Korea, depression screening was introduced in 2018 as part of a national health checkup for employed members of the National Health Insurance. The risk of suicide was significantly higher in the high-risk group for depression, as measured by the PHQ-9, than in the low-risk group (22).

A study by Yoon and Kim (23) found that the number of applicants with occupational mental illnesses increased approximately 1.74 times from 70 in 2008 to 122 in 2012, and the proportion of occupational mental illnesses among the occupational illnesses was 0.72% in 2008, but increased every year to 1.70% in 2012. In addition, the number of applicants for work-related suicides increased 8-fold from six in 2008 to 48 in 2012, and the proportion of applicants for work-related suicides among those with occupational diseases increased from 0.06% in 2008 to 0.64% in 2012 (23).

According to data on industrial accident decisions released by the Korea Labor Welfare Corporation, the number of workers who applied for industrial accidents due to mental illness in Korea increased from 331 in 2019 to 581 in 2020 and 720 in 2021. The approval rate of workers’ compensation has also been increasing year by year, soaring to 67.9% from 34.3% in 2014. The most common conditions were adjustment disorders, followed by depression, post-traumatic stress disorder, acute stress disorder, and anxiety disorders.

Unlike physical illnesses, mental illnesses are not easily visible and are often unrecognized by those around the affected employees, as well as by the employees themselves. Therefore, it is necessary to periodically evaluate mental health. In 2018, a depression screening test was introduced during the national health checkup for employees enrolled in the National Health Insurance. The test was conducted every 10 years for people aged 20–70 years. At the time of its introduction, it was conducted for 40–70-year-olds, but as depression among young people has become a social problem in recent years, the test was expanded to include 20–30-year-olds in 2019. However, there are limitations to checking and managing workers’ mental health on a regular basis by conducting the screening only once every 10 years. Therefore, it is necessary to complement job stress assessments for organizations and individual mental health; however, no study has investigated the association and correlation between job stress and depression using questionnaires. This study examined depression-screening tests commonly used in primary healthcare from multiple perspectives.

This study had several limitations. First, there are sex-related differences in mental health and the small sample size of women did not allow for stratified analyses. However, the KOSS-SF scoring system was different and was used in all analyses. Based on this information, it is necessary to increase the sample size in future studies. Second, the study did not reflect occupational characteristics such as experience, shift work, and long working hours. Therefore, it is necessary to consider occupational characteristics in future studies.

## Conclusion

The study found that the CES-D and PHQ-9 were both associated with job stress factors; however, the CES-D was not associated with job control, whereas the PHQ-9 was not associated with job demands or relationship conflicts. Both measures were correlated with all job stress factors. Moreover, the PHQ-9 showed a higher correlation than the CES-D. To manage job stress in the workplace, the first step is to evaluate the current status of job stress factors in an organization and to assess the mental health of workers in relation to these factors. Based on the results of these evaluations, organizations should work to lower job stress and provide therapeutic interventions for high-risk groups. This study recommends the use of the KOSS-SF to evaluate job stress factors in organizations. A questionnaire that showed a high correlation with job stress factors in this study should be selected to evaluate and manage the mental health of individual workers for more effective job stress management in the workplace. In summary, this study emphasized the importance of evaluating job stress factors in the workplace and assessing the mental health of workers to manage job stress effectively. This study also recommends the use of the KOSS-SF and a specific questionnaire to evaluate and manage job stress.

## Data Availability

The raw data supporting the conclusions of this article will be made available by the authors, without undue reservation.
